# Apnoea suppresses brain activity in infants

**DOI:** 10.1162/imag_a_00236

**Published:** 2024-07-17

**Authors:** Coen S. Zandvoort, Anneleen Dereymaeker, Luke Baxter, Katrien Jansen, Gunnar Naulaers, Maarten de Vos, Caroline Hartley

**Affiliations:** Department of Paediatrics, University of Oxford, Oxford, United Kingdom; Department of Development and Regeneration, University Hospitals Leuven, Neonatal Intensive Care Unit, KU Leuven, Leuven, Belgium; Department of Development and Regeneration, University Hospitals Leuven, Child Neurology, KU Leuven, Leuven, Belgium; Department of Electrical Engineering (ESAT), STADIUS Center for Dynamical Systems, Signal Processing and Data Analytics, KU Leuven, Leuven, Belgium

**Keywords:** neonate, brain, electroencephalography, respiration, early development

## Abstract

Apnoea—the cessation of breathing—is commonly observed in premature infants. These events can reduce cerebral oxygenation and are associated with poorer neurodevelopmental outcomes. However, relatively little is known about how apnoea and shorter pauses in breathing impact brain function in infants, which will provide greater mechanistic understanding of how apnoea affects brain development. We analysed simultaneous recordings of respiration, electroencephalography (EEG), heart rate, and peripheral oxygen saturation in 124 recordings from 118 infants (post-menstrual age: 38.6 ± 2.7 weeks [mean ± standard deviation]) during apnoeas (pauses in breathing greater than 15 seconds) and shorter breathing pauses between 5 and 15 seconds. EEG amplitude significantly decreased during both apnoeas and short breathing pauses compared with normal breathing periods. Change in EEG amplitude was significantly associated with change in heart rate during apnoea and short breathing pauses and, during apnoeas only, with oxygen saturation change. No associations were found between EEG amplitude changes and apnoea/pause duration, post-menstrual age, or sleep state. As apnoeas often occur in premature infants, frequent disruption to brain activity may impact neural development and result in long-term neurodevelopmental consequences.

## 1 Introduction

Worldwide, 15 million infants are born prematurely each year (WHO, 2023), putting them at increased risk of long-term neurodevelopmental disability. Apnoea frequently occurs in preterm infants, affecting more than half, and all infants born before 29 weeks’ gestation (Eichenwald et al., 1997;Finer et al., 2006). These events can result in cerebral deoxygenation (Choi et al., 2021;Horne et al., 2018) and recurrent episodes of apnoea have been associated with poorer long-term neurodevelopmental outcomes (Janvier et al., 2004;Pillekamp et al., 2007). Yet, it remains unclear the extent to which apnoea is causative or simply correlated with poorer later-life outcomes (Williamson et al., 2021). Understanding the short-term impact of apnoea on the developing infant brain will shed light on this relationship. A comprehensive framework of the interaction between brain activity and apnoea is lacking but pivotal for gaining a better mechanistic understanding of how apnoea affects brain development in premature infants.

To date, a small number of studies have investigated the effects of apnoeic episodes on brain activity recorded using electroencephalography (EEG) in infants (Usman et al., 2023). Although the amplitude of EEG activity decreases in some apnoeic episodes compared to periods of normal breathing (Bridgers et al., 1985;Deuel, 1973;Low et al., 2012;Wulbrand & Bentele, 1994), the overall findings of these studies are inconsistent (Usman et al., 2023). Limited sample sizes (i.e., <10 infants) have precluded investigation of factors which may modulate the effects and explain these inconsistencies (Usman et al., 2023). Whilst long apnoeic episodes can cause complete suppression of cortical activity and severe hypoxia (Low et al., 2012), duration may not be associated with the level of EEG suppression for shorter episodes (Fenichel et al., 1980). Suppression of cortical activity early on in life is associated with altered development (Wikström et al., 2012), potentially inhibiting maturation and impacting cortical plasticity (Ranasinghe et al., 2015). In a rodent model of hypoxic-ischemic brain injury, suppressed EEG activity is associated with changes in neurotransmitter expression and reduced structural development (Ranasinghe et al., 2015). However, while the effect of prolonged hypoxia on the developing brain has been relatively well studied in both human and animal work, whether cortical activity consistently decreases during apnoeic episodes remains to be studied. Moreover, it remains unclear how levels of physiological instability (e.g., oxygen desaturation and bradycardia) come into play. In piglets, brain activity and oxygen saturation decreased following experimentally induced apnoea, but heart rate only decreased later (Sanocka et al., 1988). A similar pattern, with later occurring bradycardia compared with changes in EEG, was observed in a single human neonate recorded byDeuel (1973). Brain activity is modulated by changes in the concentrations of oxygen and carbon dioxide in the brain, as evidenced in animal models (Ala-Kurikka et al., 2021;Lee et al., 1996;Pospelov et al., 2020). Yet, the relationship between apnoea-related EEG changes and bradycardia and oxygen desaturation in humans requires further investigation. Finally, sleep state and the infant’s age may play a role in modulating any affect. Preterm infants spend most of their time sleeping and most apnoeas happen during active sleep, which is a state that requires most metabolic energy (Lehtonen & Martin, 2004). The EEG changes rapidly with age in preterm infants (André et al., 2010), which may predispose to different EEG changes during apnoea. Whether and how these factors lead to different EEG changes during apnoea remains to be seen.

We aimed to study the acute effects of apnoea on EEG activity in infants and its relationships with heart rate change, oxygen saturation change, breathing pause duration, age, and sleep states. Following earlier reports, we hypothesised that EEG amplitude decreases during apnoea and shorter pauses in breathing. We build on these previous results by applying a quantitative approach to characterise the modulation of cortical activity during breathing pauses using time-frequency-amplitude analysis, and by examining how cortical activity changes relate to physiological instability, pause duration, age, and sleep stages. Apnoea is often defined as cessation of breathing which lasts at least 15 seconds, or longer than 10 seconds when accompanied by bradycardia or cyanosis (Blackmon et al., 2003;Elder et al., 2013;Martin et al., 2004). We investigated how EEG activity changes in response to apnoeic episodes of at least 15 seconds in duration. Moreover, since the apnoea definition of 15 seconds is somewhat arbitrary, shorter pauses in breathing may elicit changes in neurophysiology and so we also investigated changes in EEG activity following breathing pauses between 5 and 15 seconds (from here onwards referred to as short breathing pauses). For clarity, we examined changes in EEG activity during isolated pauses in breathing only. This is opposed to periodic breathing/clustered pauses where several short breathing pauses occur closely together.

## 2 Material and Methods

### 2.1 Participants and study design

The dataset was acquired at the neonatal intensive care unit of the University Hospitals Leuven, Belgium. The study protocol was in accordance with guidelines on Good Clinical Practice and Declaration of Helsinki and approved by the ethics committee of the University Hospitals Leuven. Data were recorded for research purposes (Pillay et al., 2018,^2020^). Parents or legal guardians provided written and oral consent for their child’s data to be used for research purposes. Infants were included in the current study if they had both respiration and brain activity recordings. Data comprised a total of 124 recordings from 118 infants (112 babies had a single test occasion, and six babies had two test occasions). Demographic details are provided inTable 1.

**Table 1.** Demographic details.

Factor	
Gestational age at birth (weeks)	30.43 ± 2.90 [24 ^+4^ ; 40 ^+0^ ]
Post-menstrual age at recording (weeks)	38.56 ± 2.66 [31 ^+2^ ; 47 ^+5^ ]
Chronological age at recording (weeks)	8.13 ± 3.94 [0 ^+4^ ; 22 ^+1^ ]

Reported values are mean ± standard deviation [range]. Superscripts indicate number of days.

### 2.2 Data acquisition

EEG was recorded at a sampling rate of 250 Hz using Brain RT OSG Equipment (Mechelen, Belgium). A 9-channel full-scalp configuration comprising Fp1, Fp2, C3, Cz, C4, T3, T4, O1, and O2 electrodes (positioned according to the international 10–20 system) was used to collect EEG data in 122 out of 124 recordings. In two recordings, subsets of four and five channels were used. Data were referenced to channel Cz. EEG data were recorded for an average of 15.63 ± 6.23 [1.31; 25.96] hours (median ± interquartile range [range]).

Respiration, heart rate, and peripheral oxygen saturation were simultaneously acquired using Philips IntelliVue monitors. Respiration was recorded at the thorax (*n*= 124), abdomen (*n*= 114), and nasal airways (*n*= 103). All respiratory signals were sampled at 25 Hz. Electrocardiogram (ECG)-derived heart rate and oxygen saturation signals were acquired at 1 or 5 Hz in 111 recordings (*n*= 47 recordings at 5 Hz and*n*= 64 at 1 Hz, values were calculated by the monitor from the ECG and photoplethysmography respectively). Oxygen saturation was measured with a bandwidth of 60–100% with the sensor placed peripherally on the foot or hand.

### 2.3 Data analysis

All analyses were performed in MATLAB (version 2022b; MathWorks Inc., Natick, USA) and are summarised inFigure 1. In brief, we first identified apnoeas and shorter pauses in breathing from respiration signals. Next, we investigated changes in EEG amplitude during apnoea and shorter pauses in breathing compared with periods of normal breathing using time-frequency analysis. Finally, we compared the change in EEG amplitude with change in heart rate, change in oxygen saturation, duration of the breathing pause, age of the infant, and sleep states.

**Fig. 1.**
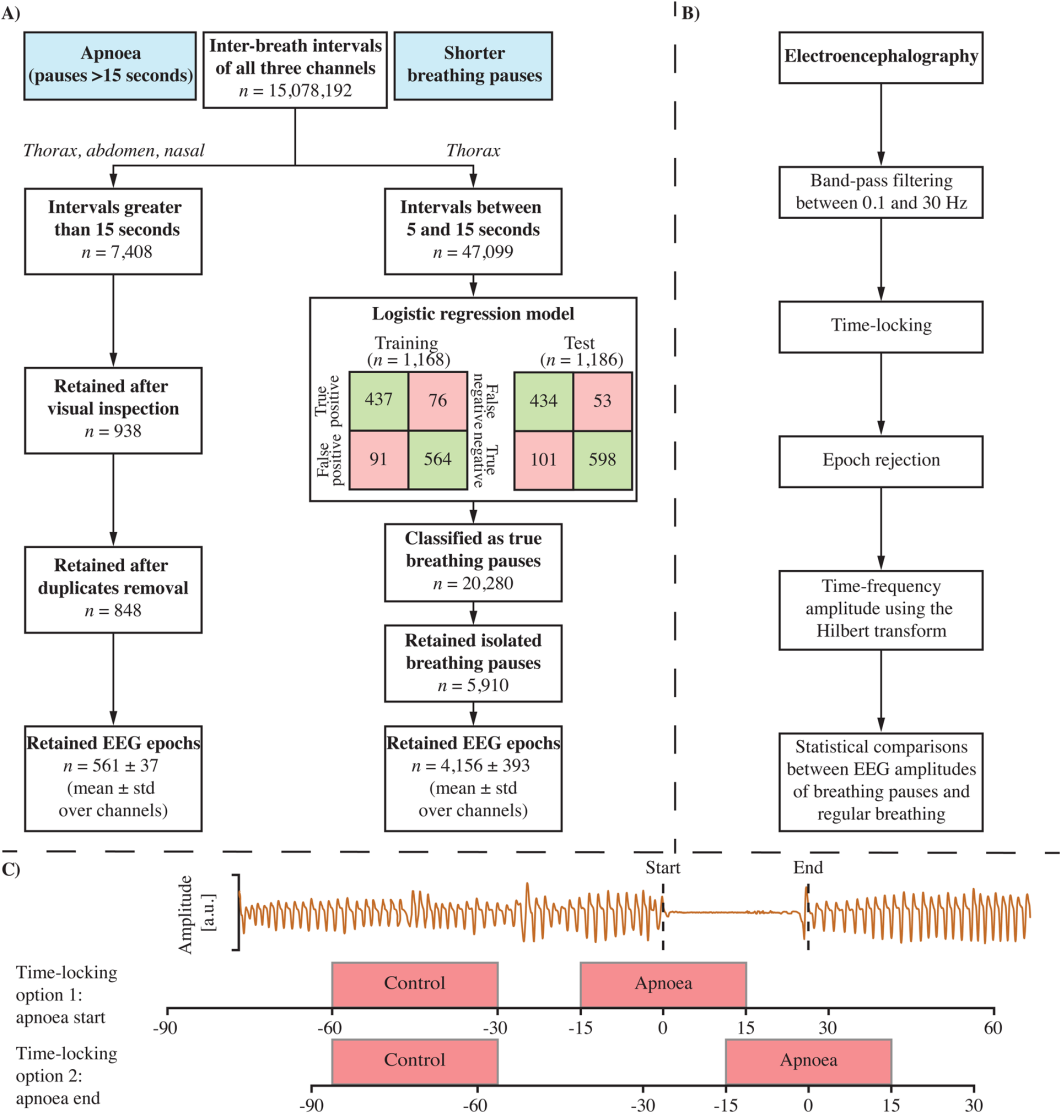
Flowchart of breathing pause identification and electroencephalographic analysis. (A) Breakdown is provided for apnoeas (pauses greater than 15 seconds; left stream) and short breathing pauses (between 5 and 15 seconds) (right stream). (B) Analysis pipeline of the electroencephalography. (C) Example of a respiratory signal containing an apnoea and showing the two time-locking options used in the analysis. Option 1: the EEG was aligned to the apnoea start, option 2: the EEG was aligned to the apnoea end. The control period was a period of the same duration in the baseline (from -90 to -15 seconds before the apnoea) when the infant was breathing normally (defined as the window in which the maximal inter-breath interval was lowest in the baseline).

### 2.4 Breathing pause identification from the respiration signals

Figure 1Aprovides an overview of the steps taken for the identification of apnoea and short breathing pauses.

Respiratory signals were first high-pass filtered with a cut-off frequency of 0.5 Hz (bidirectional second-order Butterworth filter). After that, signals were checked for amplitude outliers. Samples that exceeded five times the standard deviation of the signal were removed and linearly interpolated, a process that was iterated 10 times. We then identified the inter-breath intervals (IBI) in each of the three respiratory signals separately using a previously described algorithm validated for the identification of IBI in infants from the impedance pneumograph (Adjei et al., 2021), adapting this algorithm for the respiratory signals recorded here. Briefly, the algorithm first removes artefacts from the respiration signals that could falsely be detected as breathing cycles. It then computes a threshold according to 0.4 times the signal’s standard deviation across the previous 15 breaths. Visual validation of the algorithm on the alternative respiratory signals used here led us to modify these parameter values to a threshold of 0.5 times the standard deviation over 120 breaths. Individual breaths are detected as times when the respiratory signal crosses this threshold. Finally, the published algorithm removes false positive apnoeas using a machine-learning classifier. Due to the different signals used here, we instead visually inspected all possible apnoeas (IBI >15 seconds) to discard periods of low-amplitude signal falsely detected as apnoeas, for example, due to hypopnea/shallow breathing or artefact.

For short breathing pauses, we focused on the IBI identified from the thoracic signal, which had a higher signal-to-noise ratio than the abdomen and nasal airflow signals. From this signal, we identified 47,099 potential breathing pauses. As manually looking through each possible breathing pause is too time demanding, we developed a classification model that accurately identifies true pauses in breathing. Short breathing pauses that were identified by the classifier as a true pause in breathing were included in the EEG analysis. To derive the classification model, 1,168 possible pauses were used as a training set, and 1,186 possible pauses were used as a test set. An experienced researcher (CZ) labelled each of the possible pauses as true or incorrectly identified (e.g., due to shallow breathing or artefact). We next computed the mean absolute and standard deviation of the thoracic signals in three time windows and forwarded this as input to the classification model. Time windows consisted of the 10 to 1 seconds before and 1 to 10 seconds after the pause in breathing. The third window was from 1 second after the start of the pause in breathing to 1 second before the end of the pause in breathing. To account for amplitude differences across infants, infant-specific thoracic signals were normalised to the standard deviation of the full recording. Using the researcher’s manual ratings of the training set, a logistic regression model was trained yielding a balanced accuracy of 86% for the training set using leave-one-infant-out cross-validation. Applying this model to the test set gave a balanced accuracy of 87% demonstrating the applicability of the model to independent data. From all 47,099 potential pauses, the logistic regression classified 20,280 of them as true. To rule out any effect of short pauses in breathing in the baseline or post-pause period, we focused our analysis on isolated breathing pauses, meaning that any pauses that had another pause in temporal proximity were discarded from the analysis (here, defined as any other pause of at least 5 seconds within -60 to 90 seconds relative to the pause). This meant that we retained 5,910 isolated pauses to be included in the EEG analysis.

Taken together, a total of 848 apnoeas were detected in 64 recordings with an average duration of 17.68 ± 5.22 [15.04–66.48] seconds (median ± interquartile range [range]; SupplementaryFig. S1A). Recordings contained an average of 4 ± 15 [1–105] apnoeas and infants were aged between 32 and 48 weeks, with most infants older than 36 weeks (SupplementaryFig. S1B). In 118 recordings, we identified 5,910 isolated short breathing pauses (between 5 to 15 seconds) with a median duration of 6.04 ± 1.56 [5.04–14.96] seconds (SupplementaryFig. S1C). We found 47 ± 37 [1–152] short breathing pauses per recording, and infants were aged between 32 and 48 weeks (SupplementaryFig. S1D).

### 2.5 Time-frequency EEG amplitude

We next computed the time-frequency EEG amplitude for each of the apnoeas and short breathing pauses (seeFig. 1B). To do so, we first pre-processed the EEG time series. Recording-specific EEG time series were band-pass filtered between 0.1 and 30 Hz (bidirectional second-order Butterworth filter). We limit our spectral analysis to 30 Hz as infant EEG does typically not encompass higher frequencies such as the gamma band (>30 Hz) (André et al., 2010). EEG time series were then epoched from -90 to 150 seconds around the apnoea and from -90 to 90 seconds around the short breathing pause onset. We chose 150 and 90 seconds after the pause onset because of the different pause durations; this allowed us to have 60 seconds of data after the pause ended. We next inspected the data quality of these EEG epochs, which was done visually for apnoeas and automated for the short breathing pauses. Apnoea-related EEG epochs were visually inspected for artefacts including gross movement artefact and electrical interference and were rejected on a channel-wise basis (561 ± 37 epochs were retained; see SupplementaryFig. S2for some example time series from different babies). For the short breathing pauses, we rejected epochs with excessive amplitudes, here defined as values below -500 or higher than 500 µV (4,156 ± 393 epochs were retained).

For each epoch, we computed the time-frequency EEG amplitude. To do this, EEG data were iteratively band-pass filtered at centre frequencies of 1.5 to 29.5 Hz with steps of 1 Hz (i.e., 1.5, 2.5, 3.5,…, 29.5 Hz) and cut-off frequencies at -1 and 1 Hz around the centre frequencies (second-order bidirectional Butterworth filter). Band-pass filtered EEG time series were transformed to the analytic signal using the Hilbert transform, which contains amplitude and phase information in the form of a complex-valued signal. We took the modulus of this analytic signal to retain the amplitude. Time-frequency-amplitude representations were logarithmically transformed before averaging aiming to bring the distribution closer to being Gaussian (Halliday et al., 1995).

### 2.6 Normal breathing

We aimed to test whether the time-frequency EEG amplitude was different between apnoeas/short breathing pauses and periods of normal breathing. To define the latter, we searched for periods of normal breathing in the 90 seconds leading up to the apnoea/short breathing pause. We therefore determined the maximal inter-breath interval in time windows of either 30 seconds (for comparison with apnoeas) or 10 seconds (for comparison with short breathing pauses), shifted the window by 1 second, and computed the maximal inter-breath interval again. This was done up to 15 and 5 seconds (apnoea and short breathing pauses respectively) before the start of the breathing pause. We then selected the time window in which the maximal inter-breath interval was lowest as normal breathing. Put differently, this was the time window which had the shortest “pause” between breaths over the entire window, which was 1.16 ± 0.36 seconds (median ± interquartile range over all epochs). The selection of the normal breathing time window was done for every breathing pause individually. Time windows were set to 30 and 10 seconds as time-frequency EEG amplitudes were segmented between -15 and 15 seconds (apnoeas) and -5 and 5 seconds (short breathing pauses) at the start/end during the statistical comparisons (see below). An example apnoea and comparison normal breathing period are provided inFigure 1C.

### 2.7 Defining the heart rate and peripheral oxygen saturation outcomes

Before computing the heart rate and oxygen saturation outcomes, we first pre-processed these physiological data. Heart rate values below 40 or above 230 beats per minute and oxygen saturation values above 100% were marked and discarded from the time series. Oxygen saturation was automatically capped at a minimum value of 60% by the recording software.

Heart rate and oxygen saturation changes were defined as the change during the breathing pause relative to the time window used for normal breathing (see SupplementaryFig. S3for the absolute pre- and post-values). During the breathing pause, we selected the minimal value of the heart rate and oxygen saturation in the -5 to 60 seconds window relative to the breathing pause end. This longer time window was chosen as the grand average responses showed changes in this time window. EEG and respiratory data of recordings without heart rate and oxygen saturation were not included in this analysis (removing 13 apnoeas and 648 shorter pauses from this analysis).

### 2.8 Defining EEG-dependent sleep stages

Sleep states were identified from the EEG activity using a previously validated algorithm (Ansari et al., 2021;Ghimatgar et al., 2020;Pillay et al., 2018). Depending on the infant’s PMA, the algorithm estimated either two (<36 weeks PMA) or four (>36 weeks PMA) states. Infants below 36 weeks can be in quiet sleep (QS) or non-quiet sleep (NQS). Each of these states split into two sub-states once an infant is 36 weeks old, where QS becomes tracé alternant (TA) and high-voltage slow wave (HVS), and NQS splits into active sleep I (ASI) and low-voltage irregular (LVI). The EEG was segmented into 30-seconds epochs, and one state was assigned to each of these epochs. For the linear models (see below), we selected the epoch in which the apnoea and breathing pause was initiated.

### 2.9 Statistical analysis

We tested if the time-frequency EEG amplitudes significantly differed between normal breathing and breathing pauses (both apnoeas and shorter breathing pauses). We focused on the start and end of the pause (to account for differences in pause durations). Time-frequency EEG amplitudes were segmented between -15 and 15 seconds (apnoeas) and -5 and 5 seconds (short breathing pauses) at the start and end. Segments of breathing pauses and normal breathing were statistically compared using sample-wise within-subject*t*-tests with α-levels including false discovery rates of 0.01.

We investigated whether time-frequency EEG amplitude changes were associated with heart rate changes, oxygen saturation changes, breathing pause duration, post-menstrual age (PMA; sum of gestational age at birth and postnatal age), and sleep states. To do this, we created five linear mixed-effects models with EEG amplitude changes as response variable, one for each fixed factor. Infant was included as a random factor on both intercept and slope. More formally,

$$Y_{n}=\beta_{0}\;+\;\;\;\beta_{1}X_{n,1}+\gamma_{1}Z_{n,1}+\varepsilon_{n}$$ (1)

with$Y_{n}$being the outcome variable (EEG amplitude changes),$\beta_{0}$the intercept,$\beta_{1}$the fixed-effects regression coefficient,$X_{n,1}$the design matrix of the fixed effect (heart rate changes, oxygen saturation changes, breathing pause duration, PMA, or sleep state—each were modelled separately),$\gamma_{1}$the random-effects regression coefficient,$Z_{n,1}$the design matrix of the random effect (infant), and$\varepsilon_{n}$the residuals. Time-frequency EEG amplitude changes were calculated in the time windows of -5 to 5 seconds relative to the breathing pause end, which is where the maximal effect occurred (seeFigs. 2–4). Time-frequency EEG amplitude changes were averaged over all time and frequency samples for each apnoea/pause. For PMA, to get a single value for the EEG amplitude changes per recording, we averaged the amplitudes for every infant over all breathing pauses (after having averaged over time and frequency). This is because the number of breathing pauses will vary for every recording. Sleep stage was defined as a categorical variable with the four stages (TA, HVS, ASI, and LVI) as different levels. As the majority of infants were older than 36 weeks and so could be categorised with all four sleep stages, the statistical analysis focused on those infants, excluding five recordings who were younger than 36 weeks.

**Fig. 2.**
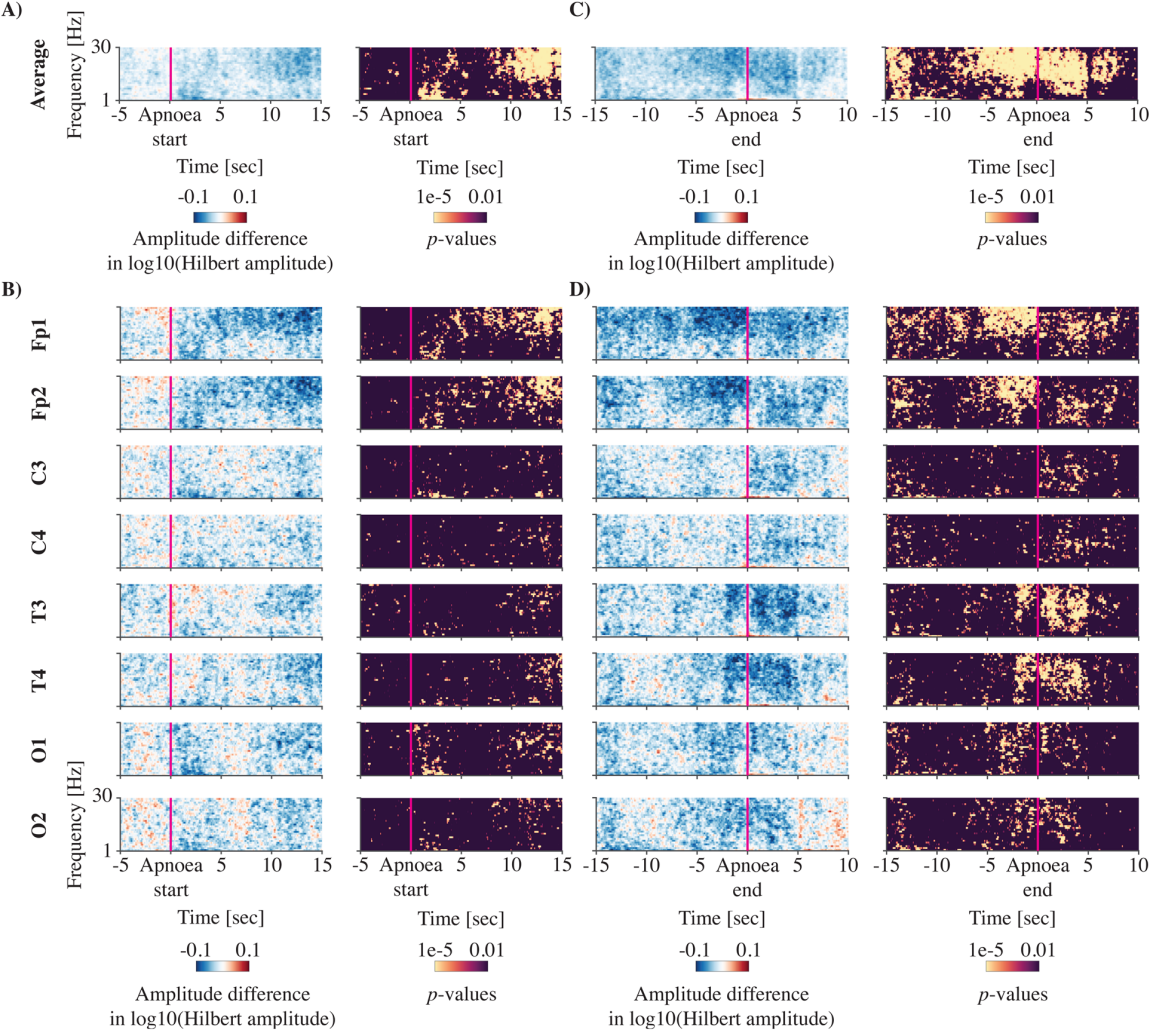
Apnoea-related time-frequency EEG amplitude changes. EEG amplitude changes across all apnoeas of at least 15 seconds in duration (n = 848 in 64 recordings). Mean EEG amplitude during the apnoea is compared to normal breathing in the time-frequency plane. Due to the varying apnoea durations, we time-locked to both the start of the apnoea (A, B) and the end of the apnoea (C, D). (A) and (C) show the amplitude change averaged across all channels, and (B**)**and (D) are channel specific. Each panel shows the amplitude difference between the apnoea and normal breathing on the left (blue and red plot) and the corresponding statistical difference at each time-frequency point on the right (yellow and black plot). A negative, blue-coloured amplitude difference means a decrease during the apnoea. Statistical significance is set to a false discovery rate of 0.01. Amplitudes are log10-transformed.

## 3 Results

### 3.1 EEG amplitude decreases during both apnoea and shorter pauses in breathing

We compared the time-frequency EEG amplitudes during apnoea and short breathing pauses with periods of normal breathing. Due to varying durations, we assessed EEG amplitude changes time-locked to both the start and end of the apnoeas and short breathing pauses. Immediately after the apnoea started, the mean EEG amplitude decreased significantly relative to normal breathing and this suppression continued during the apnoea (Fig. 2A). This change in EEG amplitude was particularly marked for frontal channels (Fp1 and Fp2) (Fig. 2B). To a lesser extent, amplitude changes were also observed at central (C3 and C4), temporal (T3 and T4), and occipital channels (O1 and O2) channels. Time-locking to the end of the apnoea, decreases in EEG amplitude were more prominent, occurred across all frequencies, and continued to occur for approximately 5 seconds after the apnoea had ended (Fig. 2C). Similar to the start of the apnoea, while the decrease in EEG amplitude was most pronounced at frontal electrodes, significant decreases in EEG amplitude were observed across all channels (Fig. 2D).

**Fig. 3.**
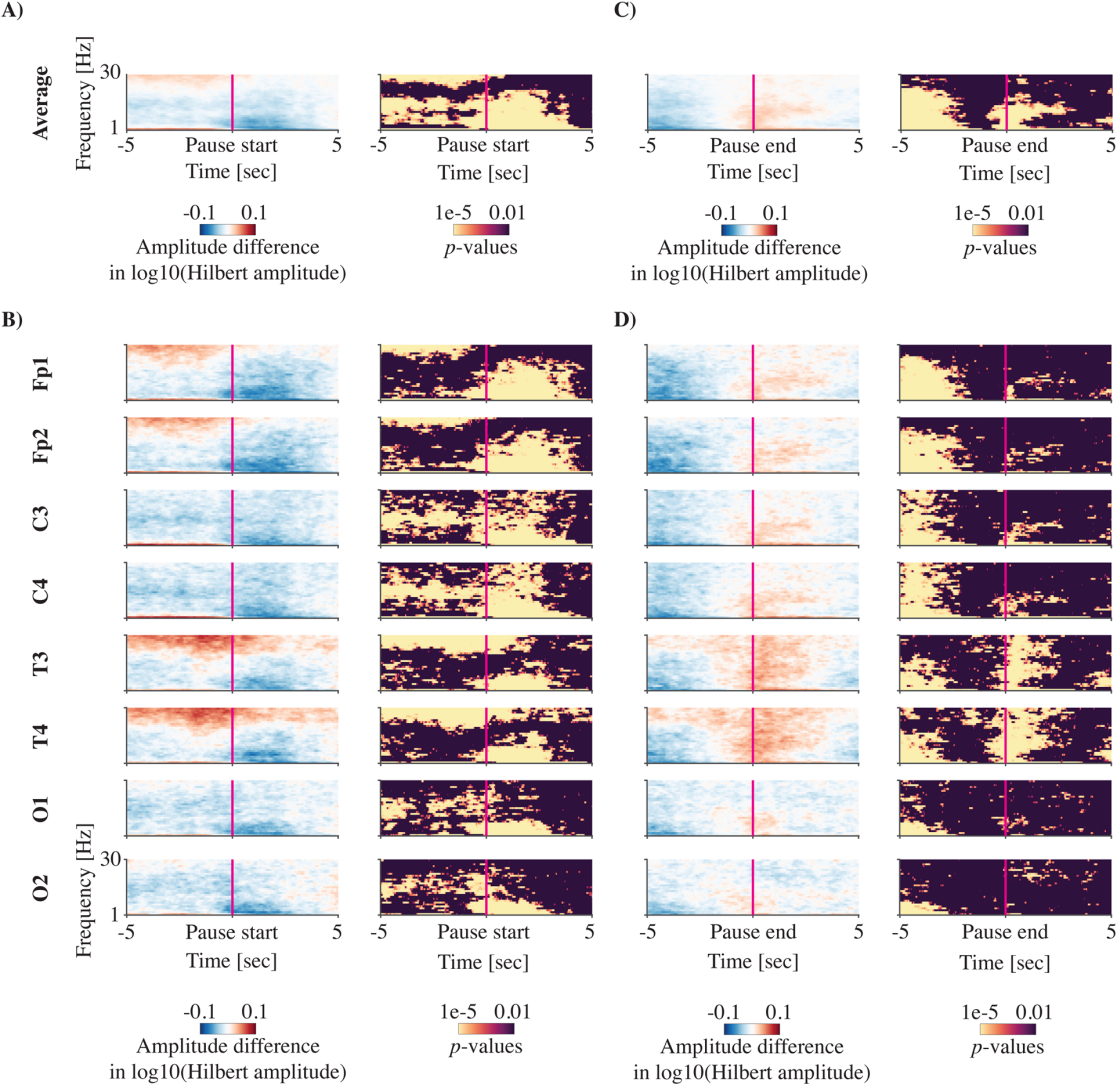
Time-frequency EEG amplitude changes during breathing pauses (between 5 and 15 seconds). Mean EEG amplitudes during the pause are compared to normal breathing for 5,910 pauses in breathing in 118 recordings. EEG amplitude changes were assessed time-locked to pause start (A, B) and end (C, D). (A) and (C) show the amplitude change averaged across all channels, and (B) and (D) are channel specific. Each panel shows the amplitude difference between the pause in breathing and normal breathing on the left (blue and red plot) and the corresponding statistical difference at each time-frequency point on the right (yellow and black plot). A negative (blue) amplitude difference means a decrease during the breathing pause. Statistical significance is set to a false discovery rate of 0.01. Amplitudes are log10-transformed. Note that due to the substantial difference in number of breathing pauses, direct comparisons withFigure 2are not possible.

Similarly, for short breathing pauses, we found that the average EEG amplitude over all channels decreased during the pause (Fig. 3A). Moreover, the 5 seconds before the pause start were characterised by a decrease at lower frequencies (~5 to 20 Hz) and increase at higher frequencies around 30 Hz, particularly at temporal channels (Fig. 3B). Immediately after the pause started, the time-frequency EEG amplitude reduced for low frequencies (typically below ~20 Hz) across all channels (Fig. 3B). Time-locking to the end of the pause, a significant decrease in EEG amplitude was observed during the pause in breathing but, unlike apnoeas, this decrease in EEG amplitude does not remain after the end of the pause in breathing (Fig. 3C, D). In fact, in the average and in some channels, there was a significant increase in EEG amplitude at the end of the pause in breathing.

### 3.2 EEG amplitude changes are related to changes in heart rate and oxygen saturation

**Fig. 4.**
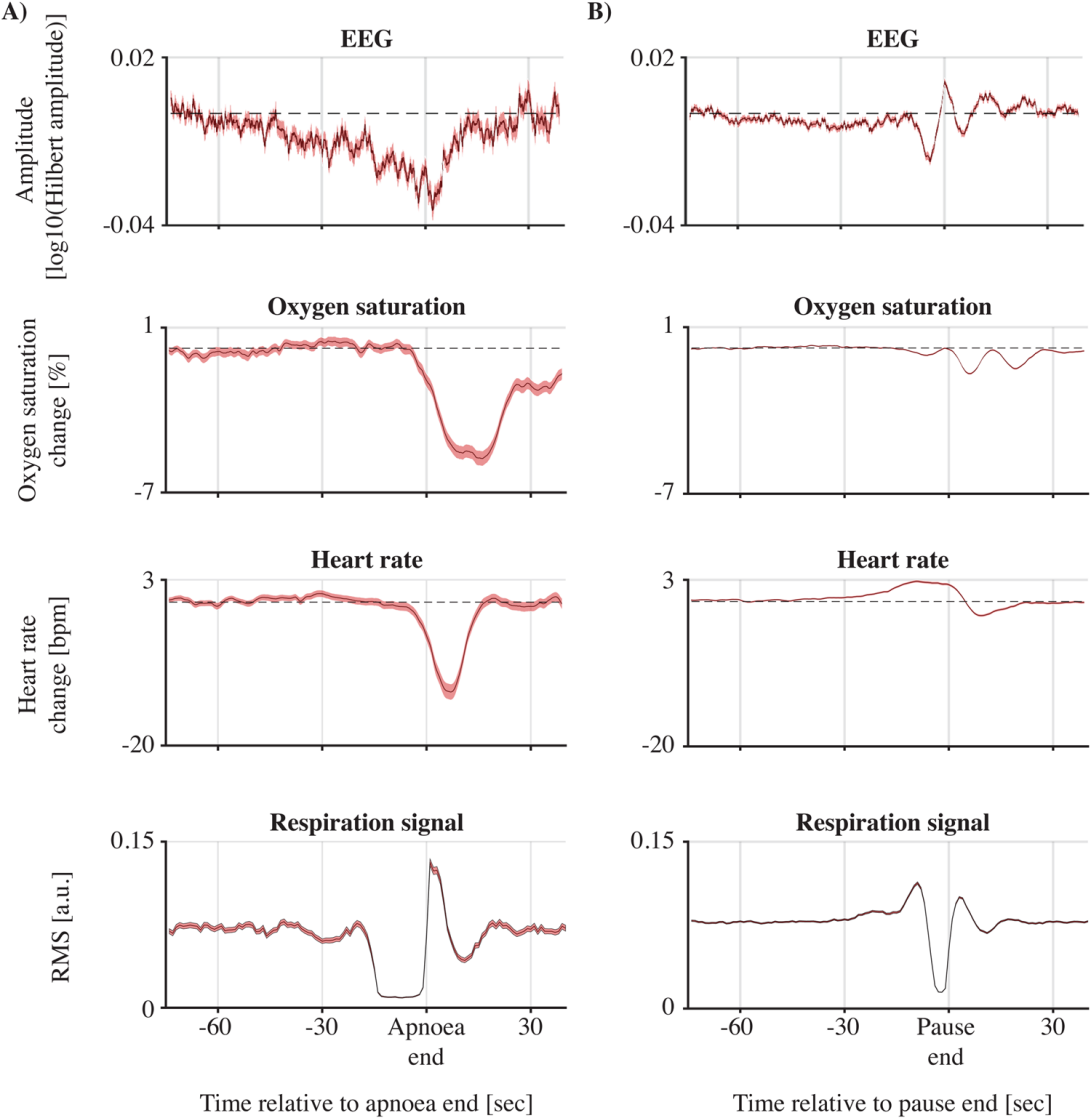
Time-resolved EEG amplitudes and vital signs. Time series were time-locked to the end of the (A) apnoeas and (B) short breathing pauses. Continuous graphs are the mean over all pauses with the shaded surfaces indicating the standard error of the mean. Time-frequency EEG amplitude and vital signs changes are the mean over all pauses (either apnoea [n = 848] or short breathing pauses [n = 5,910]) and channels. Time-resolved EEG amplitudes were averaged over frequencies and channels. The respiration signal was defined as a moving root-mean-square (RMS) in one-second windows. The moving average of each breathing pause was normalised to its own norm before averaging over breathing pauses.

**Fig. 5.**
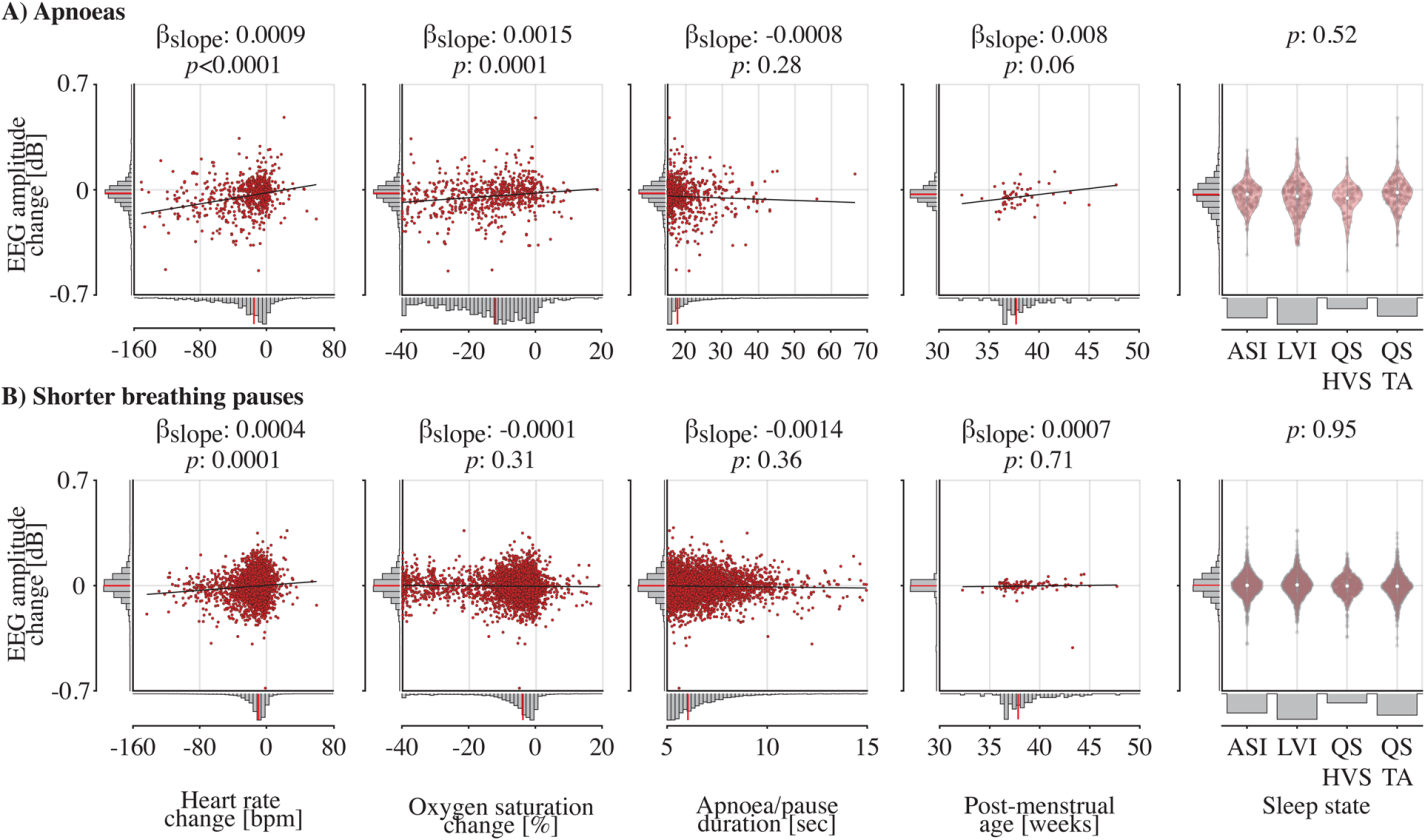
EEG amplitude changes and their associations with heart rate difference, oxygen saturation difference, short breathing pause/apnoea duration, post-menstrual age, and sleep states. EEG amplitude changes for the (A) apnoeas and (B) short breathing pauses compared with each of the predictor variables. Heart rate and oxygen saturation changes are computed as the minimal value in the time window of -5 to 60 seconds relative to apnoea and breathing pause end compared with the baseline. Baseline window was defined when the maximal inter-breath interval was lowest in the 90 seconds leading up to the pause. Red data points are the data from a single short breathing pause/apnoea in the case of heart rate change, oxygen saturation change, pause duration, and sleep state. For comparison with post-menstrual age, we averaged the amplitude changes over all pauses of a recording. The solid black graphs indicate the model fit. For the regression with sleep states, infants who were older than 36 weeks (so had four sleep states) were included (n = 119 recordings). Red bar graphs show the mean EEG amplitude change for every sleep state. For all graphs, the histograms on the x- and y-axes show the predictor and response variables’ distributions (including their median indicated by the red line).

To compare EEG amplitude changes with alterations in heart rate and oxygen saturation, we computed the time-resolved EEG amplitude by pooling the EEG amplitudes over all frequency bands and channels (Fig. 4; SupplementaryFig. S4show the time-resolved EEG amplitude for each channel separately). As expected, for both apnoea and short breathing pauses, on average heart rate and oxygen saturation decreased (Fig. 4). Change in EEG amplitude was positively associated with change in heart rate for both apnoea ($\beta_{slope}$= 0.0009;*p*< 0.0001;Fig. 5A) and short breathing pauses ($\beta_{slope}$= 0.0004;*p*= 0.0001;Fig. 5B). Change in EEG amplitude was significantly correlated with change in oxygen saturation during apnoeas ($\beta_{slope}$= 0.0015;*p*= 0.0001) but not for short breathing pauses ($\beta_{slope}$= -0.0001;*p*= 0.31). In contrast, the EEG amplitude changes did not significantly relate to either pause/apnoea duration, age, or sleep state at the start of apnoea/short breathing pause (Fig. 5). Consistent with previous research (Lehtonen & Martin, 2004), infants mostly experienced apnoeas and short breathing pauses during active sleep states (Supplementary Results andFig. S5). Moreover, in the majority of cases (over 90%), sleep state did not change throughout the apnoea (Supplementary Results andFig. S6).

## 4 Discussion

We investigated how breathing pauses alter cortical activity in infants. Consistent with our hypothesis, EEG amplitude significantly decreased during both apnoea and short breathing pauses. A reduction in cortical activity during apnoea is in line with previous studies (Bridgers et al., 1985;Deuel, 1973;Low et al., 2012;Wulbrand & Bentele, 1994). While most of these studies have reported how cortical activity changes based on relatively small sample sizes (i.e., <10 infants), we demonstrated that these results are consistent in a much larger sample size. Moreover, changes in EEG amplitude were significantly associated with changes in heart rate and oxygen saturation but not apnoea/pause duration, the age of the infant, or sleep state.

To reduce apnoeic episodes, infants are typically administered methylxanthines such as caffeine. Such pharmacological interventions have an antagonising effect on adenosine receptors, thus blocking the inhibitory effect of adenosine in the brain and leading to neuronal excitability (Mitchell & MacFarlane, 2020). In contrast, adenosine is rapidly released during hypoxic periods, which often occurs during apnoea. Hypoxia may thus suppress the generation of postsynaptic potentials and reduce neuronal excitability (Ilie et al., 2006). In the macroscopic sense, this would appear as a reduction in cortical activity, which is in line with the current results. There are suggestions that this inhibition of neuronal activity may be neuroprotective (Low et al., 2012), and also relate to changing carbon dioxide levels. Hypercapnia is associated with restoring the brain’s oxygen partial pressure and protecting the brain against metabolic acidosis (Pospelov et al., 2020) and leads to reduced neuronal excitability (Ala-Kurikka et al., 2021). This may be to avoid metabolic failure and irreversible cell damage in the brain (Nabetani et al., 1995).

Frequently reduced EEG amplitude may still have a negative impact on brain development. Blocking, reducing, or changing the patterning of brain activity can disrupt development as emphasised in animal and computational models (Gailus et al., 2021;Hartley et al., 2020). Rodent models showed that periods of decreased brain activity lower glutamatergic signalling in the cortex (Ranasinghe et al., 2015). This may lead to an inhibition of cortical layer maturation by, for example, slower dendrite development (Ranasinghe et al., 2015) and altered formation of synaptic connectivity, as suggested in computational models (Hartley et al., 2020). If episodes of apnoea frequently occur and lead to changes in brain activity, this may result in poorer neurodevelopmental outcomes (Janvier et al., 2004;Pillekamp et al., 2007;Poets et al., 2015).

Importantly, we also observed changes in cortical activity before and immediately after the breathing pause started. This EEG suppression is unlikely to be due to oxygen and carbon dioxide changes which likely rely on slower dynamics (Fig. 4). Apnoea of prematurity is believed to be the result of a variety of factors, including immaturity of the autonomic nervous system (Martin et al., 1986). The central autonomic nervous system, located in the brainstem, has direct connections with the cerebral cortex (Jänig, 2008), which are already established in infants (Schneebaum Sender et al., 2017). We postulate that the EEG amplitude changes around the apnoea onset are the result of this autonomic dysregulation. Future research should examine whether the autonomic nervous system changes (e.g., heart rate variability) and EEG suppression are coupled by quantifying connectivity between both. This notion can be further extended by including respiratory and heart rate signals, where the latter may act as a mediator.

Apnoeas in infants often co-occur with bradycardia and oxygen desaturations (Poets & Southall, 1991). In our data, both heart rate and oxygen saturation decreases were apparent following apnoea and EEG amplitude changes were associated with heart rate changes and to a lesser extent with oxygen saturation changes. The correlation with change in heart rate is in contrast to a previous study (Curzi-Dascalova et al., 2000). However, this study included only five infants with 77 isolated pauses in breathing and compared heart rate and EEG frequency, rather than amplitude, changes. Bradycardia may be directly caused by the hypoxic effect on the heart during an apnoea (Girling, 1972;Henderson-Smart et al., 1986). When severe, it may also elicit a decrease in cardiac output, cerebral blood flow, and systemic blood pressure. The latter is not well developed in the infant brain (Weindling & Kissack, 2001) and may hence result in cerebral hypo-perfusion. This may contribute to the change in brain activity during the pause in breathing. In our data, we cannot dissociate whether the EEG suppression is due to the bradycardia (and decrease in cardiac output/cerebral blood flow) or the combination of apnoea and bradycardia. Interestingly, the rate of cerebral blood flow changes may be region dependent which is shown in animal studies investigating asphyxia (Stonestreet et al., 1982). There, blood flow decreased more rapidly in rostral compared to caudal areas of the central nervous system (Greisen, 1992), which may be why we observed that the EEG amplitude changes were most profound in the frontal cerebral areas. Indeed, this may partly underlie the channel-specific frequent patterns we found (e.g.,Fig. 2Dsuggests that frontal and temporal channels have stronger higher frequency changes than the central and occipital channels). Such area- and frequency-specific changes warrant further investigation.

To a lesser extent than with heart rate, we observed positive associations between EEG amplitude changes and oxygen saturation changes (only for apnoeas and not for short breathing pauses). However, a limitation of our recording of oxygen saturation is that the minimum value was truncated at 60%, meaning that the changes may have been even larger than the maximal change of 40%. Moreover, studies using near-infrared spectroscopy (NIRS) have found that, during apnoea, changes in peripheral oxygenation are not always correlated with changes in cerebral oxygenation, particularly for less severe events (Watkin et al., 1996), and that in many cases cerebral oxygenation is maintained above 60% (Schmid et al., 2015). This is due to vasodilation occurring following rising carbon dioxide and lowering oxygen levels and highlights a limitation of our work in that we did not record changes in carbon dioxide levels. Future work should combine EEG and NIRS recordings during apnoea, in addition to measuring blood carbon dioxide levels, to further study this mechanism.

Consistent with a previous study (Fenichel et al., 1980), we did not find a relationship between breathing pause duration and EEG amplitude changes. Although changes in physiology are correlated with the duration of an apnoea, there is wide variation, with some comparatively short apnoeas leading to profound oxygen desaturations and bradycardia (Upton et al., 1991). We also did not find a relationship between EEG amplitude changes and age. While the youngest infant included in the study was 31 weeks PMA, the majority of the infants were studied when they were above 36 weeks. Although not significant, there was a trend towards greater amplitude suppression during apnoea in infants of younger ages and so further investigations in this area including younger premature infants are needed. Finally, we focused our analysis on changes in EEG amplitude during isolated pauses (with no other pauses from -60 to 90 seconds after the pause). This enabled us to clearly attribute changes in EEG activity to the single pauses and compare with other changes in physiology. However, clusters of pauses in breathing, such as in periodic breathing, can occur frequently in preterm infants, and future work should study EEG activity during clustered pauses.

In summary, we found that cortical activity decreases during breathing pauses over the whole cortex. This modulation of cortical activity was observed for both apnoeas (pauses greater than 15 seconds) and shorter breathing pauses. Although the decrease in cortical activity may not be harmful, it is unknown if this extrapolates to repeated apnoeas which result in chronic intermittent hypoxia, and how this affects long-term brain development. This warrants further investigation in future studies. Moreover, our study mostly focused on term-corrected infants. Due to the rapid functional and structural changes in the preterm period, neurophysiological effects may be larger in preterm infants and further investigation may shed light on critical developmental windows for the effects of apnoea on brain development.

## Supplementary Material

Supplementary Material

## Data Availability

The data that support the study findings are available upon reasonable request. Data sharing requests should be directed toanneleen.dereymaeker@uzleuven.be. Requests for code sharing should be directed tocaroline.hartley@paediatrics.ox.ac.uk. The algorithm to determine the inter-breath intervals from the impedance pneumographic signal is available at:https://gitlab.com/paediatric_neuroimaging/identify_ibi_from_ip.git. Usage instructions can be found at:https://doi.org/10.1136/bmjresp-2021-001042. The main codes for the EEG and physiology analysis of this paper can be found at:https://github.com/CoenZandvoort/Apnoea_EEG_term_infants/.
