# Supplementary material for: Apnoea suppresses brain activity in infants
Source: Imaging Neurosci (Camb). 2024 Jul 17;2:imag-2-00236. doi: 10.1162/imag_a_00236 (PMC12272192; doi:10.1162/imag_a_00236)
Supplement: Supplementary Material [file imag_a_00236-supp.pdf]

Supplementary Material for:

Apnoea suppresses brain activity in infants

Coen S. Zandvoort, Anneleen Dereymaeker, Luke Baxter, Katrien Jansen, Gunnar Naulaers, Maarten de Vos, Caroline Hartley

Supplementary Results

Breathing pause dependency on sleep stages

Sleep stages could be classified into two sleep states for infants below 36 weeks post-menstrual age (PMA) and four sleep stages for infants older than 36 weeks. In both age groups, the majority of apnoeas happened during non-quiet (active) sleep stages (NQS; Figure S5A). Of the 38 apnoeas in infants below 36 weeks (five recordings), almost all were during non-quiet sleep (92.11%). In the older infants (119 recordings), the percentage of apnoeas during non-quiet sleep was lower but still represented the majority (combined percentage of 61.99% in the two active sleep stages: active sleep I [ASI; 26.21%] and low-voltage irregular [LVI; 35.78%] for the 805 apnoeas). The probability of staying in the same sleep stage during and after the apnoea was around 0.9 (Figure S6A).

Similarly, short breathing pauses primarily occurred during non-quiet sleep (Figure S5B) and the probability of staying in the same sleep state during and after the short breathing pauses was higher than 0.9 (Figure S6B).

Supplementary figures

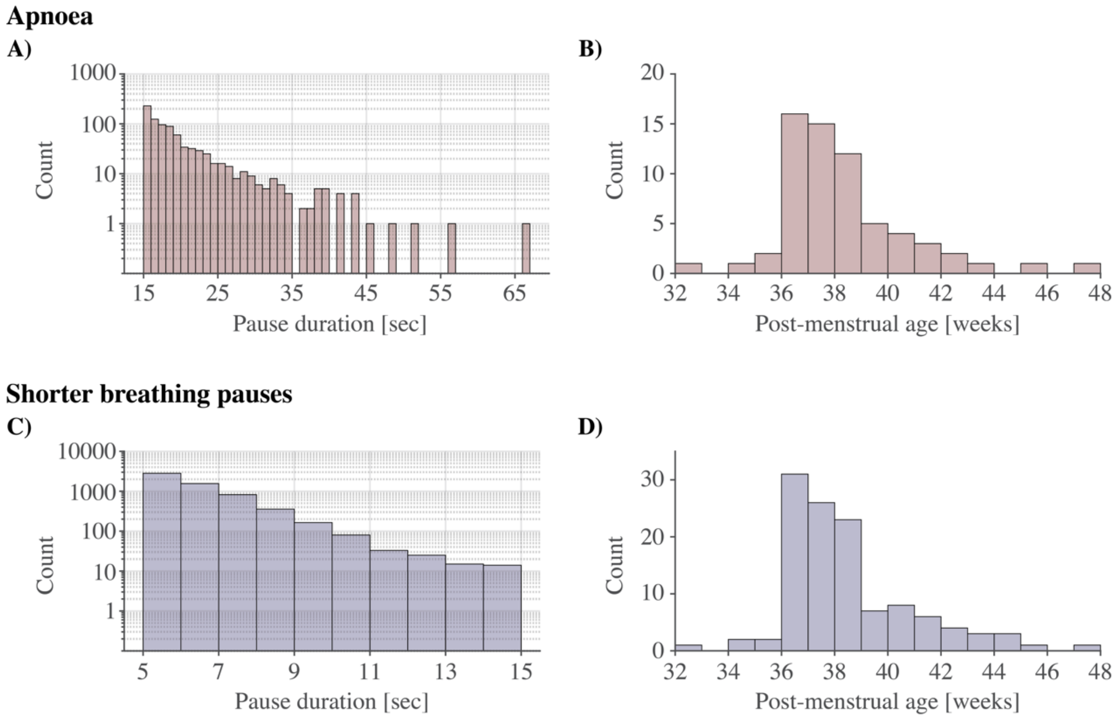

Figure S1. Apnoea/breathing pause durations and post-menstrual ages of infants experiencing cessation of breathing. *Number of* ***A)*** *apnoeas as a function of the pause duration (shown on a logarithmic scale to improve visualisation) and* ***B)*** *post-menstrual ages at time of the recording of infants with apnoeas (n = 64). Number of* ***C)*** *short breathing pauses as a function of the pause duration and* ***D)*** *post-menstrual ages of infants with these short breathing pauses (n = 118).*

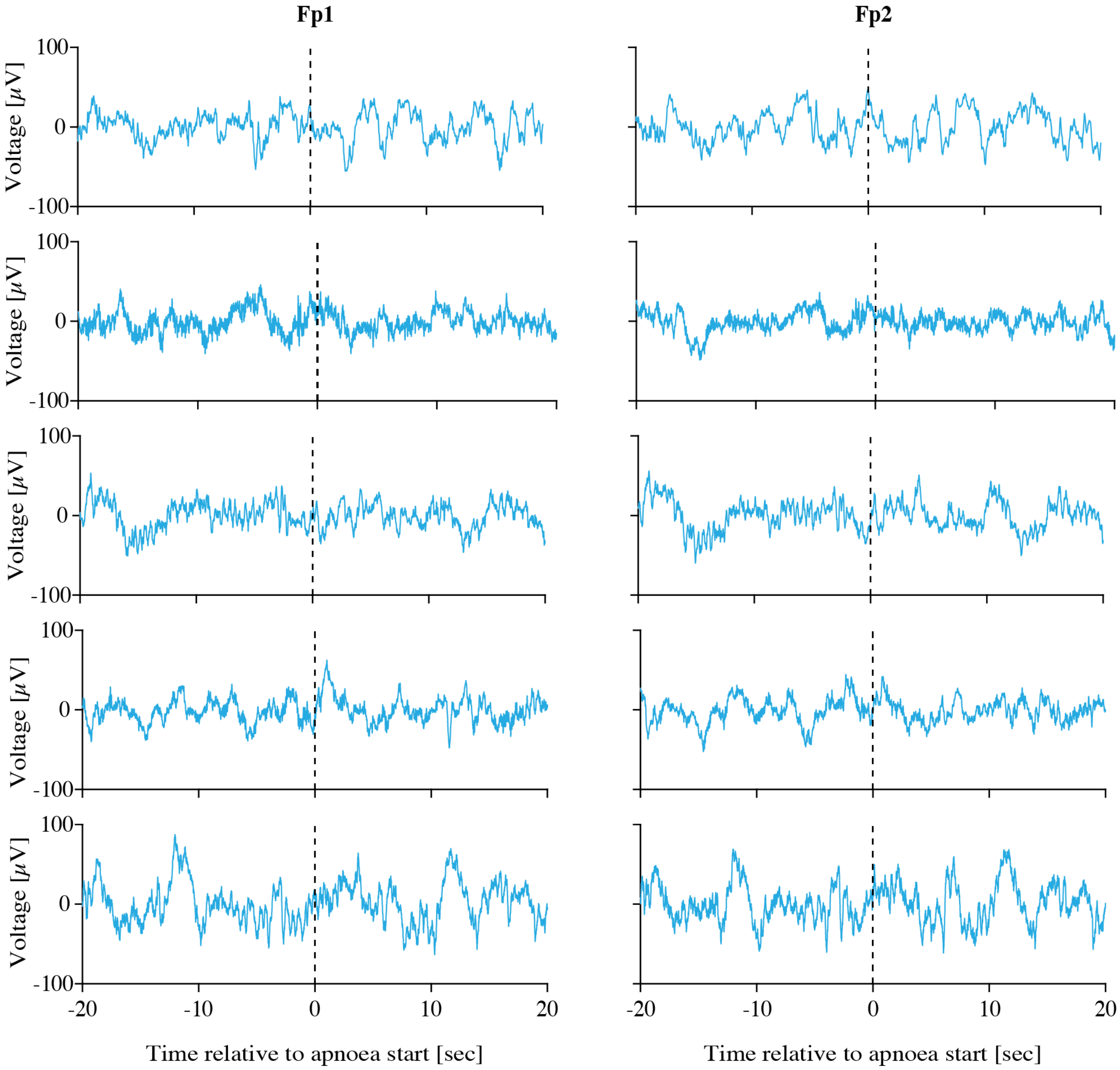

Figure S2. Illustrative EEG epochs from the two frontal channels Fp1 and Fp2. *EEG traces are from five different infants at the time they experienced an apnoea (here starting at t = 0 sec and indicated by the vertical dashed line).*

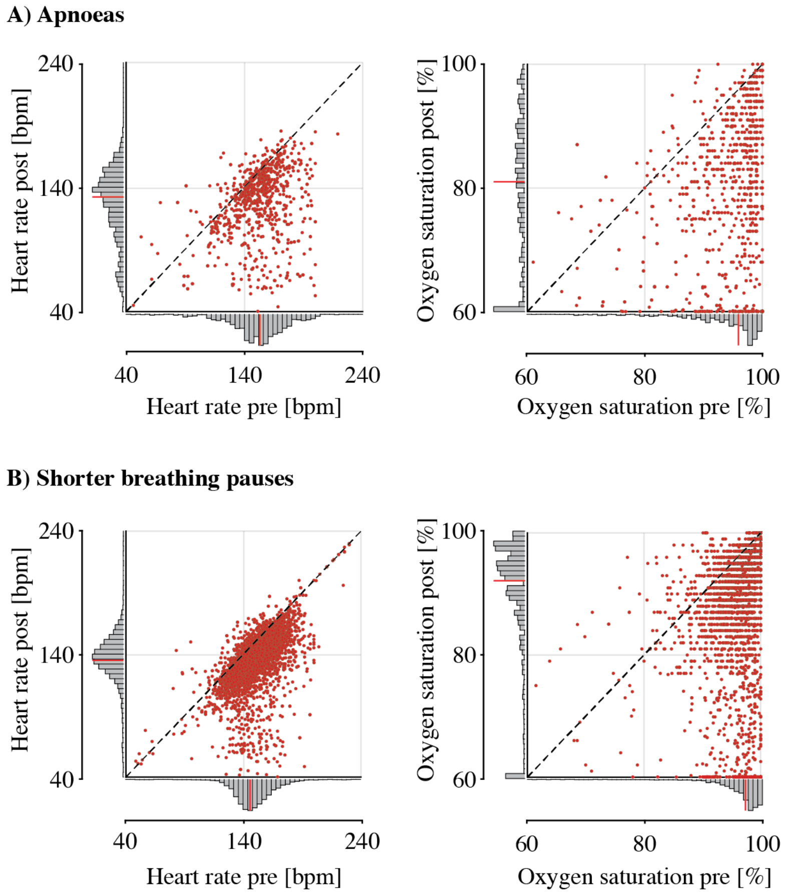

Figure S3. Absolute heart rate and oxygen saturation before and during/post A) apnoeas and B) short breathing pauses. *Red data points are the data from a single pause/apnoea. Pre-values for heart rate and oxygen saturation were taken from the time point when the maximal inter-breath interval was lowest in the 90 seconds leading up to the pause. Post-values were computed as the minimal value in the time window of -5 to 60 seconds. The dashed black graphs indicate the diagonal (i.e., pre value is equal to post value). Histograms on the x- and y-axes show the distributions of the pre and post values (including their median indicated by the red line).*

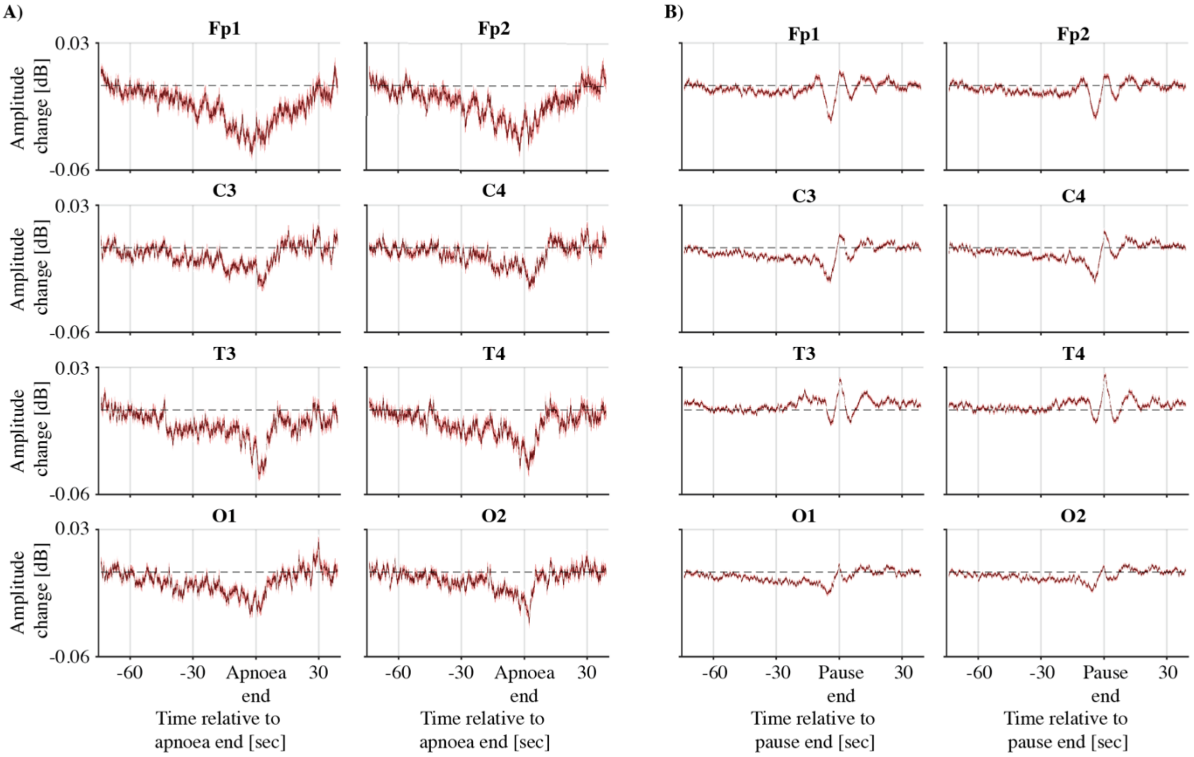

Figure S4. Time-resolved EEG amplitudes during A) apnoea and B) short breathing pauses. *Amplitudes are pooled over frequencies from 1 and 30 Hz, and time-locked to the end of the apnoea/short breathing pause. Continuous thick graphs present the mean amplitude. Shaded surfaces are standard error of the mean over apnoeas/short breathing pauses.*

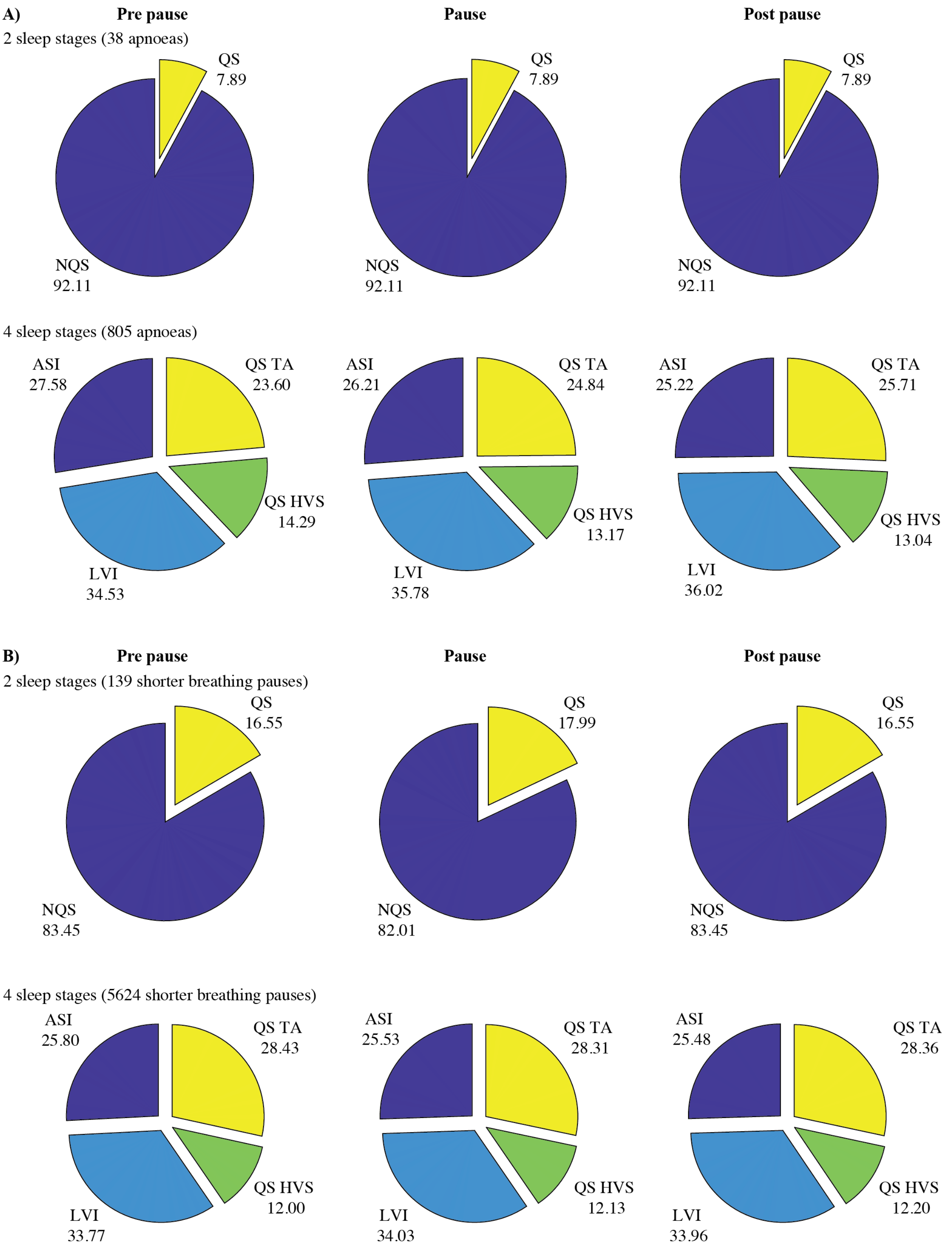

Figure S5. Sleep states before, during and after breathing pauses for A) apnoeas and B) short breathing pauses. *Number of apnoeas/pauses in each sleep stage are expressed as percentage relative to the total number of apnoeas/pauses. For infants younger than 36 weeks, two sleep stages could be extracted (NQS: non-quiet sleep and QS: quiet sleep). Both stages were divided into another two stages for infants older than 36 weeks (ASI: active sleep I; LVI: low-voltage irregular; TA: tracé alternant; and HVS: high-voltage slow wave).*

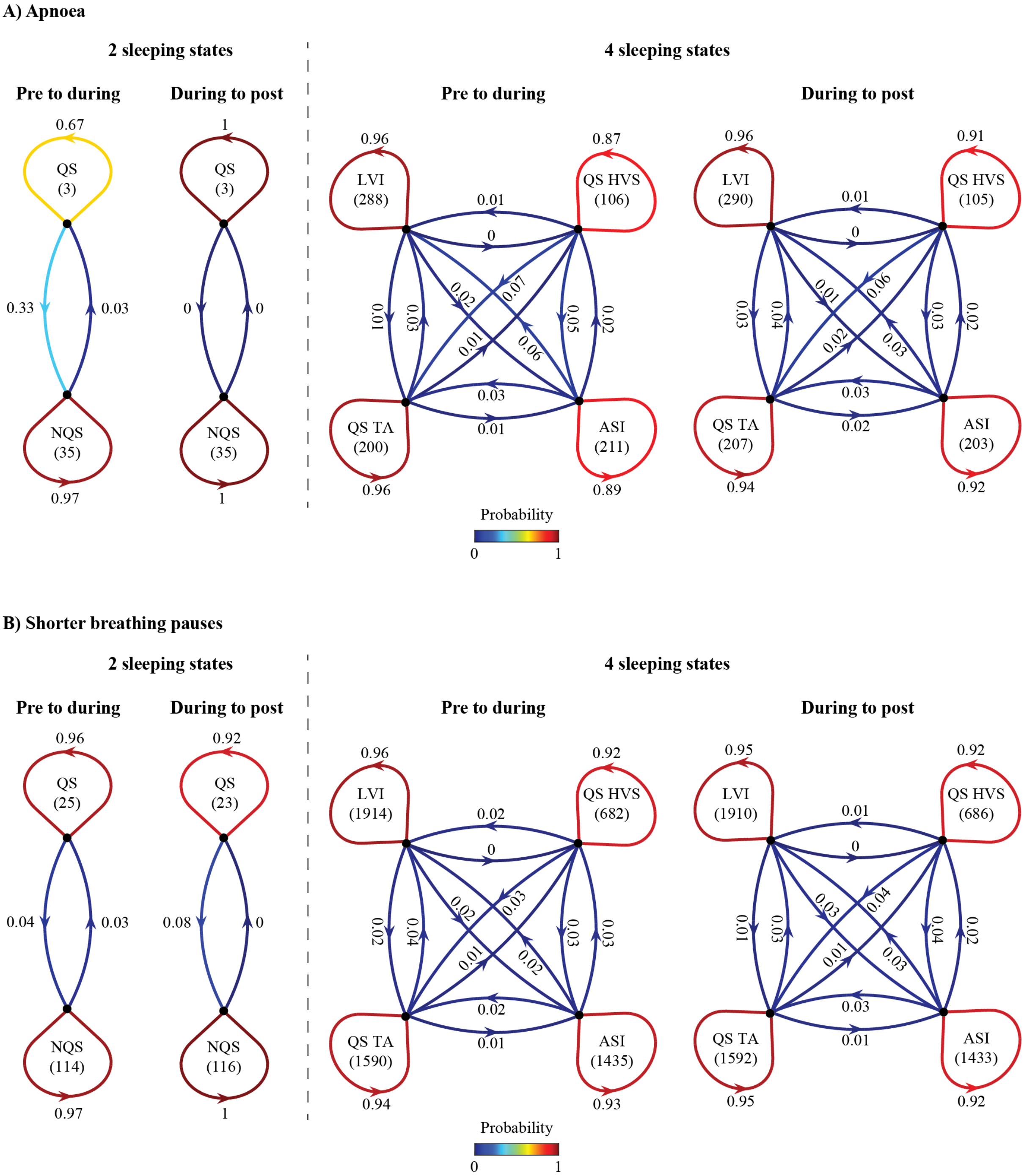

Figure S6. Sleep state transitions between 'Pre to during' and 'During to post' pause for A) apnoeas and B) short breathing pauses. *Transition matrices provide probabilities on how likely it is to switch from one sleep state to another. Absolute number below the sleep state abbreviation is the total number of apnoeas/short breathing pauses during that particular sleep state. For infants younger than 36 weeks, two sleep stages could be extracted (NQS: non-quiet sleep and QS: quiet sleep). Both stages were divided into another two stages for infants older than 36 weeks (ASI: active sleep I; LVI: low-voltage irregular; TA: tracé alternant; and HVS: high-voltage slow wave).*
